# Lincomycin Biosynthesis Involves a Tyrosine Hydroxylating Heme Protein of an Unusual Enzyme Family

**DOI:** 10.1371/journal.pone.0079974

**Published:** 2013-12-04

**Authors:** Jitka Novotna, Jana Olsovska, Petr Novak, Peter Mojzes, Radka Chaloupkova, Zdenek Kamenik, Jaroslav Spizek, Eva Kutejova, Marketa Mareckova, Pavel Tichy, Jiri Damborsky, Jiri Janata

**Affiliations:** 1 Institute of Microbiology, Academy of Sciences of the Czech Republic, Prague, Czech Republic; 2 Central-European Technology Institute, Brno, Czech Republic; 3 Crop Research Institute, Drnovska Prague, Czech Republic; 4 Institute of Physics, Faculty of Mathematics and Physics, Charles University, Prague, Czech Republic; 5 Loschmidt Laboratories, Institute of Experimental Biology and National Centre for Biomolecular Research, Brno, Czech Republic; 6 Institute of Molecular Biology, Slovak Academy of Sciences, Bratislava, Slovak Republic; University of Nottingham, United Kingdom

## Abstract

The gene lmbB2 of the lincomycin biosynthetic gene cluster of *Streptomyces lincolnensis* ATCC 25466 was shown to code for an unusual tyrosine hydroxylating enzyme involved in the biosynthetic pathway of this clinically important antibiotic. LmbB2 was expressed in *Escherichia coli*, purified near to homogeneity and shown to convert tyrosine to 3,4-dihydroxyphenylalanine (DOPA). In contrast to the well-known tyrosine hydroxylases (EC 1.14.16.2) and tyrosinases (EC 1.14.18.1), LmbB2 was identified as a heme protein. Mass spectrometry and Soret band-excited Raman spectroscopy of LmbB2 showed that LmbB2 contains heme b as prosthetic group. The CO-reduced differential absorption spectra of LmbB2 showed that the coordination of Fe was different from that of cytochrome P450 enzymes. LmbB2 exhibits sequence similarity to Orf13 of the anthramycin biosynthetic gene cluster, which has recently been classified as a heme peroxidase. Tyrosine hydroxylating activity of LmbB2 yielding DOPA in the presence of (6R)-5,6,7,8-tetrahydro-L-biopterin (BH_4_) was also observed. Reaction mechanism of this unique heme peroxidases family is discussed. Also, tyrosine hydroxylation was confirmed as the first step of the amino acid branch of the lincomycin biosynthesis.

## Introduction

**Figure 1:**
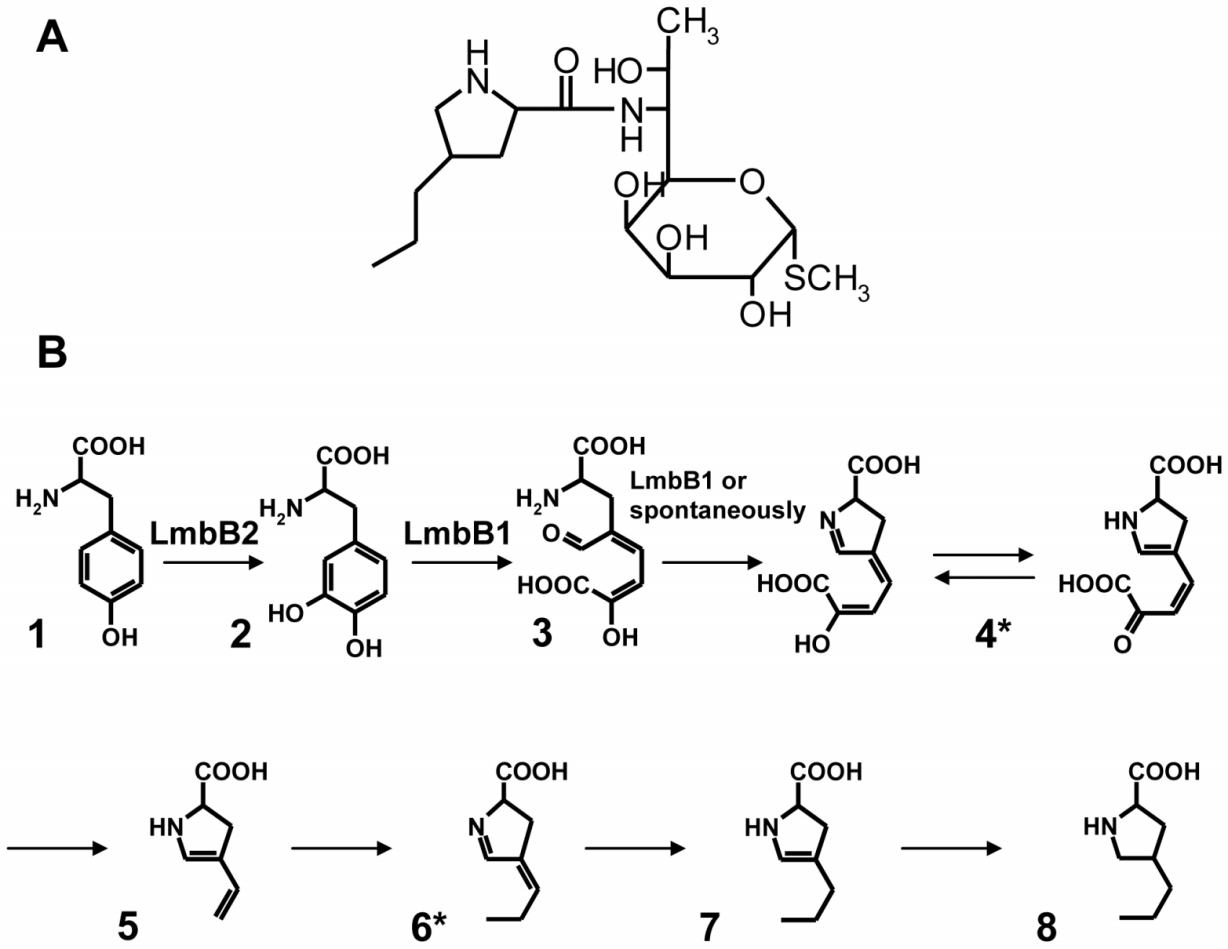
Proposed propylproline biosynthetic sub-pathway of the lincomycin (A) biosynthesis. The sub-pathway leading to propylproline formation (B) was proposed by determining the biosynthetic origin of the carbon and nitrogen atoms using feeding studies and subsequent NMR analysis [2]. Two intermediates (indicated by asterisks) were confirmed experimentally [4], [5]. The Lmb proteins known to be involved in the sub-pathway are indicated.

The clinically important antibiotic lincomycin [1] is formed by a bifurcated biosynthetic pathway. The two sub-pathways lead to 6-amino-6,8-dideoxy-1-thio-*D*-erythro-*α*-*D*-galactooctopyranoside (methylthiolincosamide) and 4-*n*-propyl-*L*-proline (propylproline). For lincomycin formation, these two products are condensed, followed by the final methylation step. This pathway was proposed based on the results of feeding studies with isotope-labeled precursors and subsequent NMR (nuclear magnetic resonance) analysis [2], [3]. However, in the sub-pathway leading to propylproline only two intermediates have so far been identified (Figure 1).

This limited knowledge of the pathway intermediates complicates the assignment of functions to the 26 ORFs (*lmb* genes) contained in the lincomycin biosynthetic gene cluster of *Streptomyces lincolnensis* ATCC25466 [6]. Of the proteins catalyzing the biosynthesis of propylproline, only the function of LmbB1 has been demonstrated experimentally. The enzyme catalyzes 2,3-extradiol cleavage of the 3,4-dihydroxyphenylalanine (DOPA) aromatic ring [5], [7]. The *lmbB1* gene is translationally coupled at its 3′ end with the *lmbB2* gene, which may indicate a functional relationship between their protein products. The function of LmbB2 in the lincomycin biosynthesis has so far only been predicted on the basis of circumstantial evidence. When *lmbB1* and *lmbB2* were co-expressed in *Escherichia coli*, enzyme activities were found which catalyzed the conversion of either *L*-tyrosine or DOPA to a yellow colored product (intermediate product 4*, Figure 1) [7]. It was hence assumed that the LmbB2 protein, either alone or in cooperation with LmbB1, may constitute an enzyme performing the *ortho* hydroxylation of the tyrosine aromatic ring. This was in accordance with the previous suggestion that the propylproline moiety of lincomycin is derived from tyrosine [2].

In bacteria, monohydroxylation of aromatic rings is generally catalyzed by monooxygenases and peroxidases that employ oxygen and hydrogen peroxide as the oxidant, respectively. The monooxygenases involve NADH dependent heme proteins cytochromes P450 [8], di-iron hydroxylases [9], and flavin monooxygenases [10], pterin or *α*-ketoglutarate dependent non-heme iron monooxygenases [11], [12], and monooxygenases with copper ions in their active centers [12]. Microbial peroxidases are generally considered to be typically extracellular enzymes involved in the degradation of recalcitrant aromatic polymers [12], however, intracellular peroxidases of two different types have recently been described to hydroxylate aromatic rings of tyrosine [13] and 3-methyl tyrosine [14] in the biosynthesis of secondary metabolites.

The deduced protein sequence of the putative tyrosine hydroxylase, LmbB2, comprises 317 AA. Database searches did not reveal any conserved domains, nevertheless, four LmbB2 orthologs located in gene clusters controlling the biosynthesis of the pyrrolo[1,4]benzodiazepine antibiotics anthramycin, sibiromycin, and tomaymycin [15], [16], [17], and hormaomycin [18] were identified. The antibiotics are biosynthetically related to lincomycin: the precursors of the proline derivatives contained in their molecules are likely to be synthesized *via* a common set of reactions comprising the hydroxylation of tyrosine to DOPA [18], [19]. Recently, the close LmbB2 ortholog found in the anthramycin gene cluster has been annotated as a peroxidase performing the hydroxylation of the tyrosine aromatic ring [13].

In the present study, the LmbB2 protein, coded for by the *lmbB2* gene of the lincomycin biosynthesis gene cluster, was expressed, purified and biochemically characterized. It was found to hydroxylate the tyrosine aromatic ring to yield DOPA. LmbB2 is a member of an unusual heme protein family. Tyrosine hydroxylating activity of LmbB2 yielding dihydroxyphenyl alanine in the presence of (6*R*)-5,6,7,8-tetrahydro-*L*-biopterin (BH_4_) was also observed. Reaction mechanism of this unique heme protein family is discussed. Also, tyrosine hydroxylation was confirmed as the first step of the amino acid branch of the lincomycin biosynthesis.

## Results and Discussion

The propyproline sub-pathway of the lincomycin biosynthesis is expected to include hydroxylation of tyrosine to DOPA (Figure 1). This activity has not yet been unambiguously assigned to a gene product of the lincomycin biosynthetic cluster; however, the LmbB2 protein has already been suggested as a candidate based on previous *in vivo* experiments [7]. In order to prove that the candidate *lmbB2* gene encodes a functional protein catalyzing the expected reaction the following experiments were performed.

### Overproduction and purification of recombinant LmbB2

LmbB2 was overexpressed in *E. coli* in the form of two different fusion proteins, linked to either maltose-binding protein or to a hexahistidyl tag. On SDS PAGE analysis, both recombinant proteins, hereafter called MBP2*-LmbB2 and LmbB2, showed the expected size of approximately 78 kDa and 36 kDa, respectively. The recombinant enzymes, MBP2*-LmbB2 and LmbB2, were purified to near homogeneity by means of maltose and metal-ion affinity chromatography, respectively.

The two fusion proteins differed in their stability. The instability index counted by help of the ProtParam software [20] classified LmbB2 as unstable and MBP2*-LmbB2 as stable. The MBP2^*^-LmbB2 protein was used for some spectrometric measurements (absorption spectrometry and resonance Raman scattering (RRS)) as it had better solubility than its LmbB2 counterpart and was stable enough after dithionite treatment to yield satisfactory spectra. His tagged LmbB2 protein was used in all other measurements. In order to prove that the data obtained using these two constructs are complementary, the MBP2^*^-LmbB2 enzyme activity was assayed and the respective spectrometric characteristics of LmbB2 protein were evaluated.

### Spectrometric analyses of LmbB

Concentrated LmbB2 solutions showed a dark red-brown color. Therefore, the LmbB2 protein was investigated by several methods to identify its prosthetic group.

**Figure 2:**
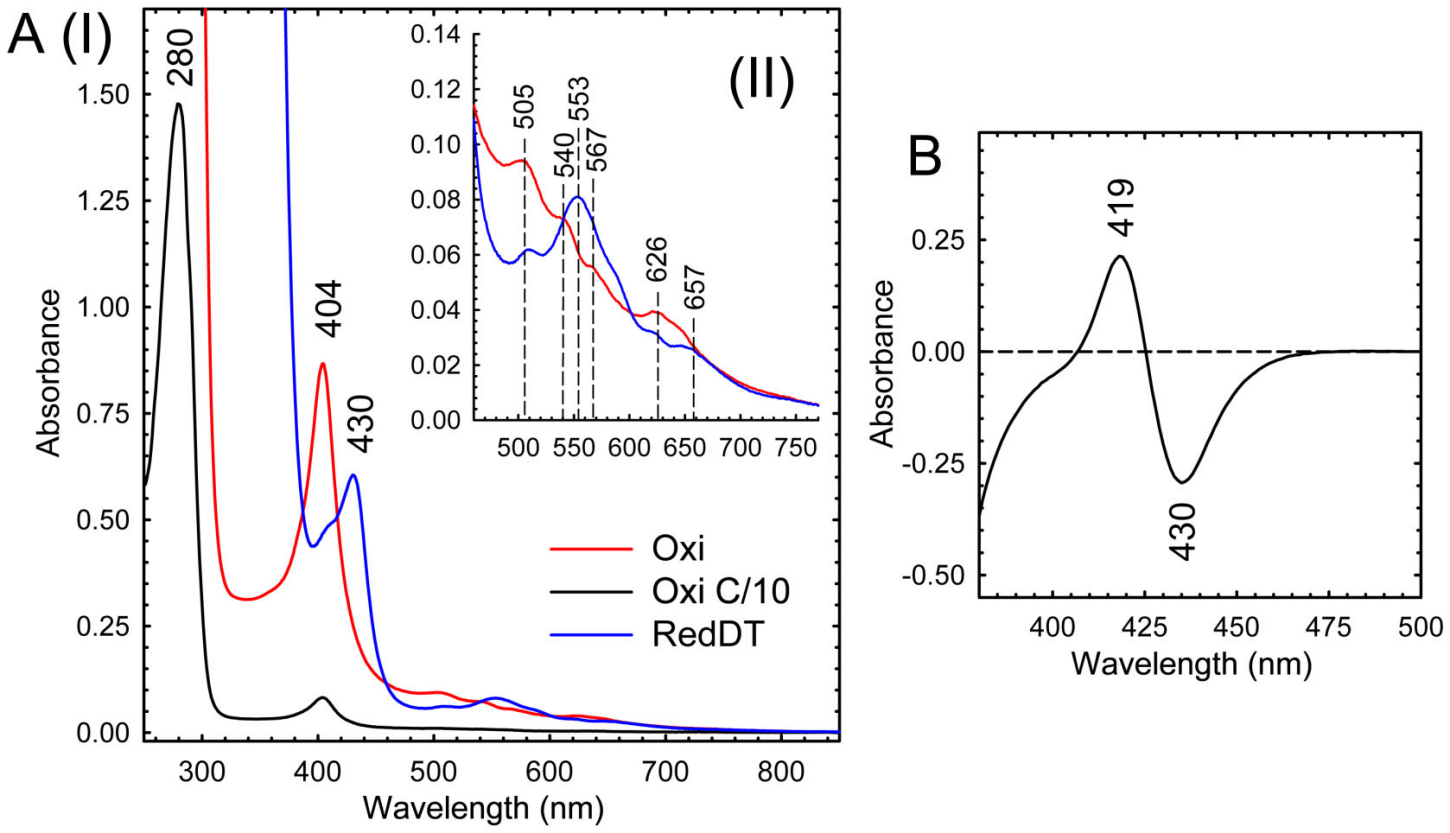
Spectrometric analysis of MBP2*- LmbB2. Absorption spectra (A) of oxidized (dashed line) and reduced (full line) forms of MBP2*- LmbB2 bore witness of heme presence in the MBP2*- LmbB2 molecule showing Soret band at 404 nm and its shift under dithionite treatment. Reduced CO-differential spectrum (B) of the MBP2*- LmbB2 did not show a maximum at 450 nm indicating that LmbB2 does not belong to the cytochrome P450 superfamily.

The UV/Vis spectrum of the purified MBP2*-LmbB2 indicated the presence of a heme prosthetic group (Figure 2A). The native enzyme had a Soret peak at 404 nm. This peak shifted to 410 after the addition of cyanide (38 mM, data not shown). The prosthetic group could be reduced upon sodium dithionite treatment (Figure 2A). The reduced-CO difference spectrum did not show a peak at 450 nm (Figure 2B), therefore, LmbB2 does not belong to the P450 superfamily.

**Figure 3:**
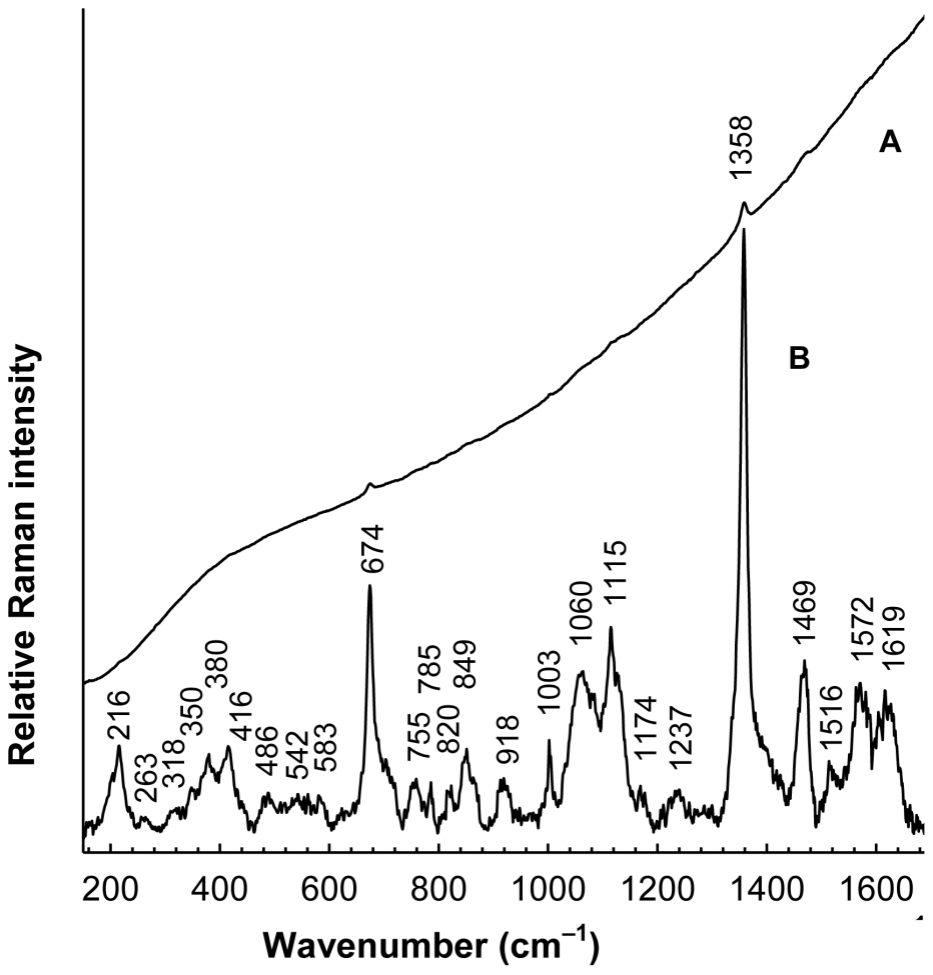
Resonance Raman spectrum of MBP2*- LmbB2. MBP2*- LmbB2 (trace A) was superimposed on a fluorescence background. Background corrected spectrum (trace B) was expanded 25× to highlight details. Upper part of the background-corrected spectrum exhibited pattern typical for the Soret band-excited RRS spectra of various heme-containing proteins [21], [22].

RRS was employed to confirm heme as the LmbB2 prosthetic group. As shown in Figure 3, Raman signal of the oxidized MBP2*-LmbB2 (background-corrected spectrum) exhibits a pattern typical for the Soret band-excited RRS spectra of various heme-containing proteins [21], [22], as demonstrated by the bands located at 1358, 1469, 1572 and 1619 cm^−1^, assignable to *ν*_4_, *ν*_3_, *ν*_2_ and *ν*_10_ and/or C = C vinyl vibration modes [21] of metalloporphyrins, respectively. Although the position of the dominant *ν*_4_ band at 1358 cm^−1^ is typical for the ferrous states [21], its occurrence in the RRS spectrum of the oxidized MBP2*-LmbB2 can be satisfactorily explained by a photoreduction upon laser irradiation of stationary samples into the Soret band, reported previously for ferric cytochrome *c* oxidase [23], [24]. Such an extensive photoreduction was first observed for the cytochrome *c* oxidase samples exhibiting strong fluorescence background and thus linked with a flavin contamination [24], nevertheless, using carefully purified flavin-free samples, the photoreduction was afterwards shown to be an inherent property of the cytochrome *c* oxidase alone [23]. Laser-induced photoreduction of the MBP2*-LmbB2 was confirmed by use of differential absorption according to methodology proposed by Ogura *et al.* [23] (Figure S1 A, B). Identification of the Fe-protoporphyrin IX as cofactor of LmbB2 can be further supported by the presence of the 216 cm^−1^ (iron-histidine stretching) and 674 cm^−1^ band found in Soret-excited RRS spectra of some heme-containing proteins [22], [25]. Moreover, two marker bands which are typical for a high spin ferrous heme protein can easily be identified at 1358 and 1469 cm^−1^. Similar marker bands positions can be found *e.g*. in the RRS of reduced horseradish peroxidase [25].

In order to specify the type of the heme group, the LmbB2 protein was inspected by UPLC (Ultra Performance Liquid Chromatography) with the subsequent HR-MS analysis (high-resolution mass spectrometry). The UPLC chromatogram (Figure S2 A) showed the peak corresponding to the LmbB2 protein (retention time 4.9 min, absorption maxima 224 and 279 nm) and, in addition, two peaks (I and II) which corresponded to two free chromophores. Peak I showed the same retention time (5.2 min) as a heme b standard which was released from catalase. Peak II showed the same retention time (6.9 min) as an authentic protoporphyrine IX standard. The absorption maxima of the corresponding peaks were 398 and 404 nm, respectively.

Peaks I and II were subsequently purified by semi-preparative HPLC and subjected to HR-MS. A signal at *m/z* = 616.17687 was observed for peak I, in accordance with the expected value for heme b (Fe-protoporphyrin IX), within 0.2 ppm error tolerance. A signal at *m/z* = 563.26489 was observed for peak II, in accordance with the expected value for protoporphyrine IX, within 0.7 ppm error tolerance (Figure S2 B).

Taken together, the LmbB2 molecule was occupied by a mixture of heme b and protoporphyrine IX. Protoporphyrine IX is most probably introduced to its molecule as a result of an incomplete heme b synthesis which is not proportional to the amount of the recombinant protein produced. Generally, study of recombinant heme proteins is accompanied by sub-optimal heme incorporation, which vary with the protein of interest and is caused by the fact that under normal physiological conditions the *E. coli* cell does not have significant levels of free heme [26]. The recombinant LmbB2 also suffered from a poor heme occupancy (approx. 10% and less). The attempt to increase the *in vivo* heme incorporation by help of addition of *δ*-aminolevulinic acid to the media was not very successful, *i.e*. a significant proportion of the recombinant sample did not contain heme. Therefore, evaluation of its enzymatic activity was systematically underestimated. Similar behavior has been observed for the close LmbB2 ortholog, Orf13, which has recently been identified as a heme protein [13].

An amino acid residue that coordinates the heme prosthetic group was not identified confidently; however, based on reduced-CO differential spectra a cysteine residue can be excluded. Moreover, the presence of the 216 cm^−1^ (iron-histidine stretching) band in the MBP2*-LmbB2 Soret-excited RRS spectra suggested that histidine could be its heme iron ligand.

Experimental data on the Ofr 13 protein also suggest histidyl ligated heme iron [13]. Pair-wise alignment between Orf13 and LmbB2 revealed that in LmbB2 a highly conserved region containing R_90_W_91_X_3_H_95_ also had the same conserved residues as the distal site RX_2_F(W)H motif of class I, II, and III peroxidases which contain a proximal histidine residue coordinating the heme iron between 120 and 138 residues away from the conserved distal histidine. In LmbB2 and Orf13 the residue spacing differs equally from that of the above mentioned heme peroxidases.

Taken together, histidine seems to be the most likely heme axial ligand in both Orf13 and LmbB2.

### Enzymatic synthesis of DOPA

When tyrosine was incubated with LmbB2, the time-dependent formation of an enzymatic product was observed by HPLC.

The retention time of 3,4-DOPA standard and the LmbB2 reaction product were identical (Figure S3 A I and A II). Moreover, the identity of the enzymatic product with 3,4-DOPA was supported by the identical UV spectra of both compounds (Figure S3 A I and II).

The LmbB2 reaction product was then purified by HPLC and analyzed by HR-MS. In the HR-MS spectrum of authentic DOPA, the signal of *m/z* 198.07613 (Δ = 0.25 ppm) corresponding to the pseudomolecular ion [C_9_H_12_N_1_O_4_]^+^ was observed. The HR-MS spectrum of the isolated LmbB2 reaction product showed the same signal at *m/z* 198.07615 (Δ = 0.35 ppm) (Figure S3 B). It could be thus concluded that the LmbB2 reaction product is DOPA.

### Effect of cofactors and reactive oxygen species (ROS) on the LmbB2 activity

**Figure 4:**
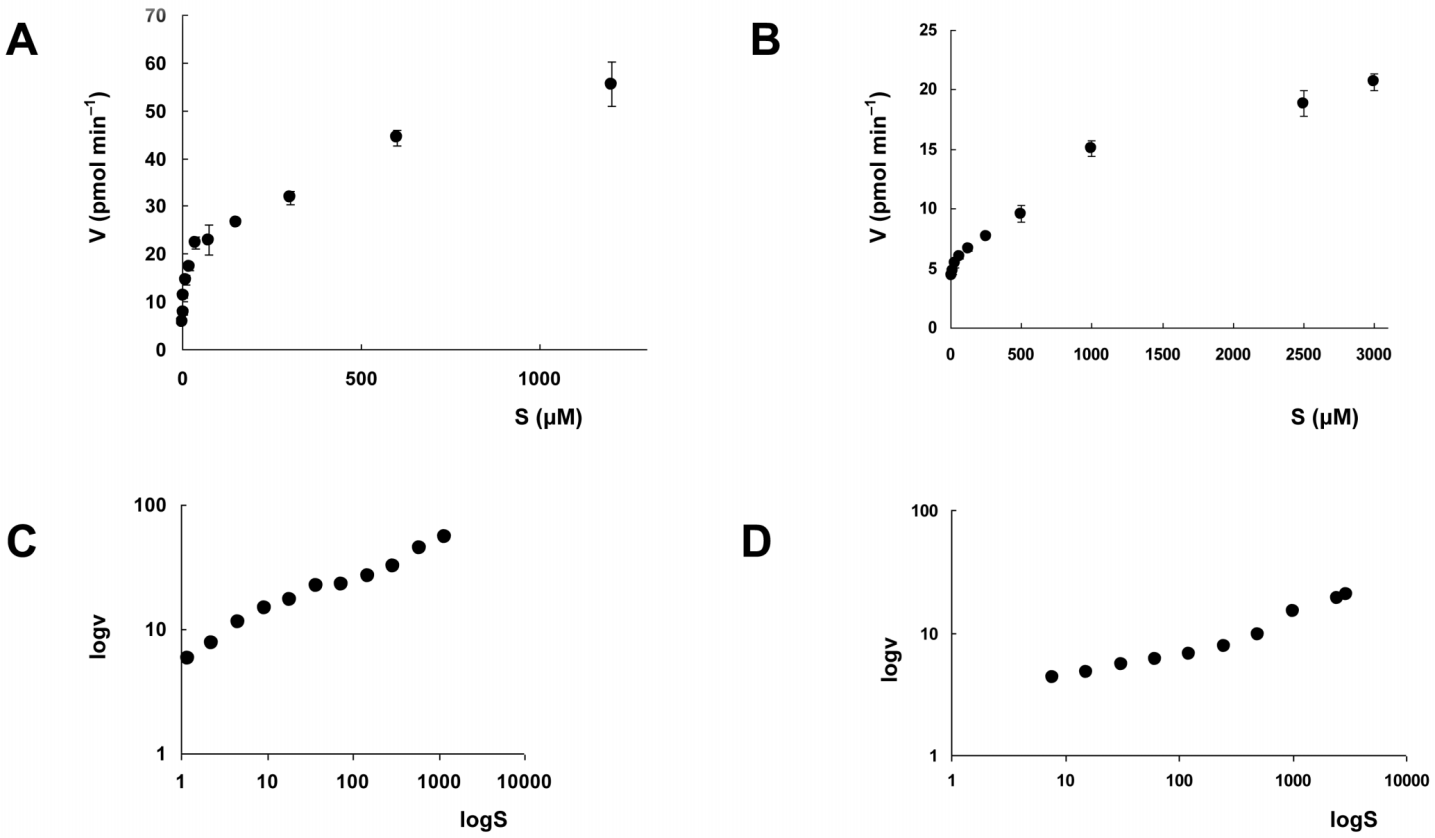
Dependence of the LmbB2 reaction rate on tyrosine/BH_4_ concentration. Dependence of the LmbB2 reaction rate on tyrosine (A) and BH_4_ (B) concentration and their respective double logarithmic plots (C, D). The velocity data represent the average of three measurements; vertical error bars represent standard deviation.

LmbB2 catalyzes DOPA synthesis from tyrosine. Nevertheless, LmbB2 behaved differently from all enzymes known to catalyze the reaction. First, LmbB2 is a heme protein. The specific activity of LmbB2 reaction correlated with the stoichiometry of heme b. The LmbB2 enriched with heme up to 35% has its specific activity 38% higher than the native protein. Moreover, interestingly, LmbB2 did not need any external oxidant or reduced cofactor to perform the reaction. Therefore, LmbB2 was inspected for the presence of tightly bound cofactors and, at the same time, the effect of addition of several reduced cofactors was tested. By virtue of the LmbB2 heme protein nature, cofactors known to cooperate with heme (and similar) have been chosen (Table 1). NAD(P)H affected the LmbB2 activity even negatively which could be explained by reduction and consequently discharge of high valent state ferryloxo intermediate similarly to P450 [27]. On the other hand, BH_4_ addition increased the activity of His tagged and MBP*tagged LmbB2 two fold and four fold, respectively. DOPA was the only hydroxylated product of the reaction and the reaction velocity increased with increased substrate concentration. However, the data did not strictly follow the Michaelis-Menten kinetics but rather testified of the fact that the reaction is not fully dependent on the enzyme catalytic cycle and a radical mediated step seemed to be included as well (Figure 4). A “combined” reaction including both an enzyme and radical step, was described *e.g*. for the system horseradish peroxidase [12], [28].

**Table 1:** Effect of metal chelators, detergents, electron donors on the LmbB2 activity.

Additive	Concentration	Relative activity (%)
None	-	100
Chelator	(mM)	
EDTA	0.1	83
	1.0	73
Detergent	(% w/v)	
SDS	0.01	85
	0.02	71
Electron donors	(mM)	
NADH*	2	75
NADPH	2	56
BH_4_ *	2	186
tetrahydrofolic acid	2	118

* compounds were tested in concentrations ranging from 2 µm to 2 mM. The results were consistent with the conclusion that BH_4_ and NADH increase and decrease, respectively, the LmbB2 activity.

**Figure 5:**
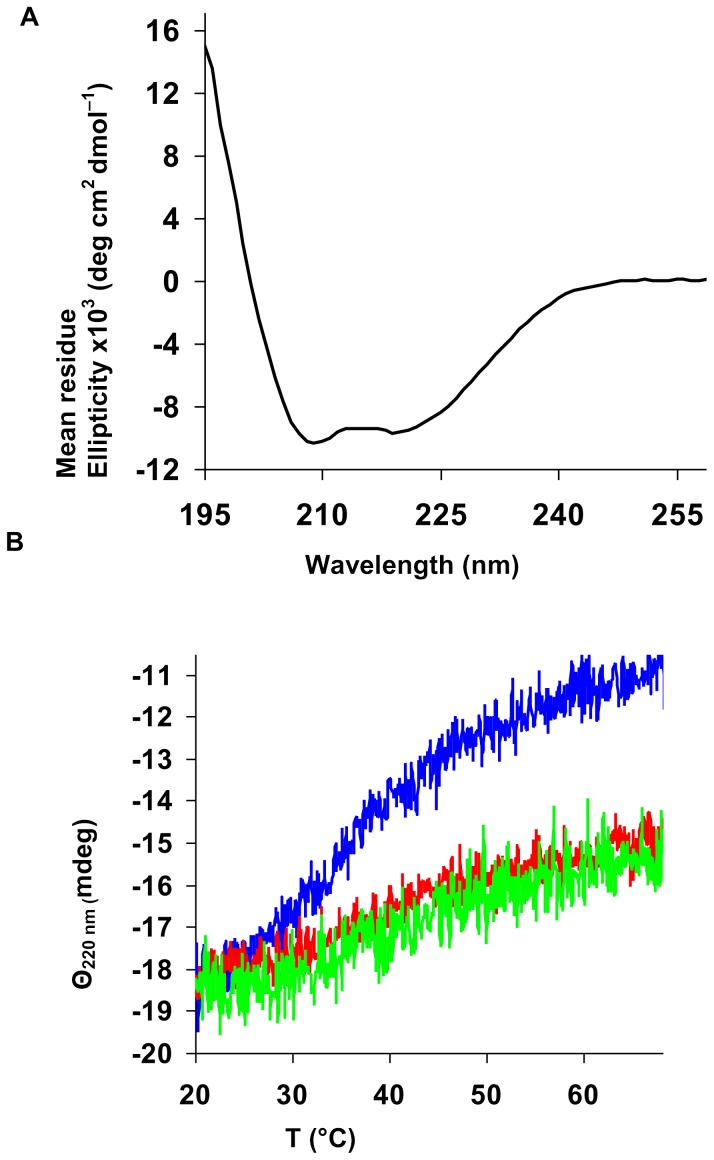
Analysis of LmbB2 by CD spectroscopy. (A) The far-UV CD spectrum of LmbB2. (B) Thermal denaturation of LmbB2 in the presence of 0 µM (blue curve), 50 µM (red curve) and 250 µM (green curve) concentration of BH_4_ bear witness of the stabilization of the LmbB2 structure by BH_4_.

Interestingly, BH_4_ also showed a positive effect on the LmbB2 thermal stability (Table 2, Figure 5). This effect cannot be simply interpreted. First possibility is that BH_4_ stabilizes LmbB2 by binding. The stabilization effect of cofactors binding to their respective enzymes has already been described. Unfortunately, a binding site similar to any of three known types of BH_4_ binding sites has not been found. Second, the stabilization effect can be mediated by reduction of the LmbB2 heme iron. Reduction of various heme proteins by BH_4_ has also been published [29].

**Table 2:** Effect of BH_4_ on melting temperature (*T*_m_) of LmbB2.

Concentration of BH_4_ (µM)	*T*m (°C)
0	35.7±0.3
50	38.0±0.3
250	39.5±0.5

BH_4_ increased the LmbB2 activity; however, the enzyme could perform the reaction without the addition of either reduced cofactors or oxidants. No tightly bound cofactor has been found in the LmbB2 molecule by help of several methods; moreover, the reaction took place even if ascorbate, used to protect DOPA from oxidation, was omitted (data not shown). Therefore, the mechanism of the LmbB2 catalyzed reaction has still been unknown.

Generally, several mechanisms other than “classical monooxygenation” used by heme proteins to hydroxylate aromates come into consideration. For example, heme proteins rarely express dioxygenase activity as their native biological fuction [30]. Heme-containing tryptophan 2,3 dioxygenase introduces two oxygen atoms into tryptophan molecule without need of any cofactor. Dioxygenation followed by elimination of water yielding DOPA cannot be excluded as the LmbB2 reaction mechanism.

Moreover Orf13, the close LmbB2 ortholog, has recently been classified as a peroxidase based on its ability to use hydrogen peroxide as an oxidant [13]. The specific activity of the LmbB2 protein is also increased in the presence of H_2_O_2_, however, is not dramatically different from that with BH_4_.

Although Orf13 was shown to produce ROS causing oxidative damage of the protein itself (in its non-protected state) it did not show activity without the addition of an external oxidant in comparison to LmbB2 [13]. Nevertheless, LmbB2 and Orf13 were treated in a different way during the purification and the respective assay and this could cause the differences in their behavior. While the LmbB2 enzyme was protected by only imidazole present in some steps during the purification and all experiments were performed with freshly prepared enzyme sample, ORF13 was protected by 50 mM dithiothreitol (DTT) during its purification and storage. Therefore, while the LmbB2 freshly desalted sample (6 mg mL^−1^) already contained a certain amount of ROS (34 µM hydrogen peroxide generated probably *via* dismutation of superoxide coming *e.g*. from side chain oxidation or ferrous heme oxidation), Orf13 was better protected from the ROS production and their role could not be evaluated. Additionally, *K*_m_ of Orf13 for hydrogen peroxide was determined with quite a high standard error (1.0 mM ±0.3) [13]. The Michaelis-Menten curves are not available, however, it can be speculated that ROS generated by the enzyme itself were present during the measurement, causing result distortion.

Surprisingly, Orf13 differs from “classical” peroxidases in several characteristics. First, heme peroxidases are irreversibly inactivated with an excess of H_2_O_2_ and in the absence of exogenous electron donors with time. This process includes the compound III formation and is accompanied by clear spectral changes [31]. Orf13 being inactivated with hydrogen peroxide did not show such a spectral change [13]. Second, heme peroxidases bind hydrogen peroxide first [32] but Orf13 was found to require pre-incubation with tyrosine [13]. This unusual substrate binding order could be explained by Orf13 treatment. ORF13 has been reduced by dithionite just prior reaction but ferric iron is a prerequisite for the peroxidase reaction [33] and DTT is considered to be a competitive inhibitor of some heme proteins [34]. Residual DTT was found for instance in the crystallized P450 active site [35] and interfered with heme modification by hydrazines [34]. Therefore, this treatment could cause the unusual substrate binding order observed for the Orf13 peroxidase reaction. Nevertheless, it seems useful to note that the mere fact that a hemoprotein is able to use H_2_O_2_ as oxidant does not necessarily mean that its natural reaction mechanism is peroxidase. For instance, cytochromes P450 are able to use H_2_O_2_ in both peroxidation and peroxygenation reaction but their native reaction mechanism is monooxygenase [27].

Interestingly, both LmbB2 and Orf13 share several common characteristics with plant seed peroxygenase. This heme imidazole protein has high spin ferric iron and labile activity and catalyzes among other hydroxylation reactions of aromatics [36]. Unfortunately, neither its reaction mechanism nor kinetics has yet been described in detail. Presently, some enzymes first described as peroxidases and nowadays mostly referred to as peroxygenases turned out to be functional hybrids that share catalytic properties with peroxidases and monooxygenases (e.g. aromatic peroxygenase, EC 1.11.2.1) [37].

Taken together, the detailed and exact determination of the real reaction mechanisms of newly described heme proteins is very complicated for several reasons. First, heme prosthetic group is extremely versatile as for the reactions catalyzed. Moreover, frequently even more than one reaction mechanism can be used by the one and only heme protein [27]. Second, not merely the proximal ligand itself but also critical structural elements at the distal active site can significantly influence the mechanism of the reaction catalyzed [38], [39], [40], [41]. The right determination of the LmbB2 heme protein is furthermore complicated by production of ROS when exposed to oxygen. It is known that some heme proteins are not only able to produce ROS but various ROS have already been established to take place in heme protein catalyzed oxidations [27], [42], [43]. In some cases they are even essential [43].

These findings thus expand the category of heme-containing enzymes which hydroxylate aromates and set the stage for further mechanistic studies.

### Biochemical characteristics of LmbB

The reduction potential of the Fe^III^/Fe^II^ couple can be influenced by pH which may affect the respective enzyme activity. Therefore, the pH optimum of the LmbB2 enzyme was investigated. The pH optimum of DOPA formation by the enzyme was 8.0 in Tris·HCl buffer and 9.0 in glycine buffer. The enzyme showed a higher activity in glycine buffer (pH 9.0) than in Tris·HCl buffer (pH 8.0), and was also more stable in the former buffer (data not shown). pH optimum of Orf13 has not been published, however, Orf13 standard assay has been performed at pH 8 [13].

Thermostability of LmbB2 was determined by assaying the enzyme activity after exposure to various temperatures for 30 min, in the absence of substrates. The enzyme lost 53% of its activity at 45°C, and nearly all of its activity at 50°C (Figure S4).

The addition of 0.1 mM bivalent metal ions (Cu^II^, Fe^II^, Zn^II^, Ca^II^ or Mg^II^) had no significant effect on the tyrosine hydroxylating activity of LmbB2 (data not shown).The activity of LmbB2 was only slightly inhibited by EDTA (10 mM). SDS decreased the LmbB2 activity (Table 1).

### Sequence analysis

PSI BLAST [44] search revealed that LmbB2 has orthologs in G^+^ and G^−^ bacteria and cyanobacteria. After four iterations, over twenty orthologs (Table S1) were identified with highly significant EXPECT scores ranging from 1×10^−107^ to 1×10^−7^ that are well below the significance threshold of 1×10^−6^ [45]. Although unusual this still growing peroxidase family seems to be widespread among bacteria.

## Conclusion

In the present study, tyrosine hydroxylation was confirmed as the first step in the lincomycin biosynthesis. The reaction is catalyzed by the LmbB2 heme protein. The LmbB2 activity is increased by BH_4_ as well as by hydrogen peroxide, however, determination of the respective reaction mechanism is considerably complicated by the production of reactive oxygen species which interferes with kinetic studies. LmbB2 belongs to a new enzyme family catalyzing aromate hydroxylation in bacteria and cyanobacteria.

## Materials and Methods

### pMAL-recombinant plasmid preparation

pTHM1 is the pGEM-3Zf(+) vector (Promega, USA) bearing the EcoRI/SacI fragment of pJN23 [7]. pTHM2 was constructed as follows: the complementary oligomers 5′-AAT TCC ATA TGA GTT CAC-3′ and 5′-TCG AGT GAA CTC ATA TGG-3′, carrying *EcoR*I, *Nde*I, *Xho*I restriction sites (underlined) were inserted between sites *Eco*RI and *Xho*I of the pTHM1.

pTHM3 is the pMAL-c2G vector (New Englad Biolabs, USA) bearing the *Eco*RI*/Hind*III fragment of pTHM2.

### pET42b - recombinant plasmid preparation

pTHH1 was constructed as follows: A ∼960 bp DNA fragment was amplified from *S. lincolnensis* ATCC25466 genomic DNA (GenBank accession number EU124663.1), using a sense primer (*Nde*I site underlined), 5′- ATA TGA GTT CAC TAG AGG CAC G - 3′ and the antisense primer (*Xho*I site underlined), 5′- CTC GAG ACT CGC CGC CGC GGT - 3′. PCR was performed in 50 µL containing template DNA (100 ng), the sense and antisense primers (0.2 pmol µl^−1^ each), dNTP (200 µM each), LA DNA polymerases mix buffer, betain (1.25 M), dimethyl sulfoxide (5%), and LA DNA polymerases mix (2.5 U, TopBio, Czech Republic). The conditions for PCR were as follows: 94°C for 2 min, then 30 cycles of 94°C for 45 s, 51°C for 45 s, and 68°C for 2 min. Final extension was carried out for 5 min at 68°C. A single ∼960 bp PCR product was obtained and subcloned into pGEM-T vector (Promega, Madison, U.S.A.) to yield pTHH1. pTHH2 is the pET42b vector (Novagen, Madison, U.S.A) bearing the *Nde*I/*Xho*I fragment of pTHH1.

### Overproduction of the recombinant protein

The variants of the LmbB2 protein were designated as follows: LmbB2 protein with maltose binding protein tag as MBP2*-LmbB2 and LmbB2 protein with histidine tag as LmbB2.

The *E. coli* BL21(DE3) cells harboring either the pTHM3 or pTHH2 plasmids were grown in LB medium with the respective antibiotics at 37°C to OD_600_ = 0.8. Overproduction was induced with isopropyl *β*-D-thiogalactoside (0.4 mM) and the cells were then incubated at 17°C for 24 hours and then harvested by centrifugation (2700 *g*, 12 min, 4°C).The pellet was suspended either in buffer A (Tris·HCl (80 mM, pH 8.0), NaCl (277 mM), glycerol (4%)) or buffer B (Tris·HCl (20 mM, pH 8.0), NaCl (200 mM), glycerol (20%)) containing lysozyme (1 mg mL^−1^), RNAse A (0.05 mg mL^−1^) and DNAse I (0.05 mg mL^−1^) for either maltose or metallo-affinity chromatography performance, respectively. The cell suspension was incubated at 4°C for 60 min and cell debris were removed by centrifugation (19000 *g*, for 25 min, 4°C).

For the maltose affinity chromatography, the cell-free extract in buffer A was diluted and loaded on a 2-mL Amylose resin column (New Englad Biolabs, USA) and MBP2*-LmbB2 protein was purified according to manufacturer's instructions. The eluted fractions containing MBP2*-LmbB2 as identified by the protein assay (Ponceau S staining on nitrocellulose membrane) were pooled and verified by SDS PAGE. The MBP2*-LmbB2 protein was then concentrated using Centricon YM-30 (Millipore, USA) and immediately used in experiments.

For metallo-affinity chromatography, the cell-free extract in buffer B was diluted and LmbB2 was purified on a 5-mL HiTrap Chelating HP column (Amersham Pharmacia Biotech, Uppsala, Sweden) according to the manufacturer's protocol. The bound protein was eluted with imidazole (300 mM) in buffer C (Tris·HCl (20 mM, pH 8.0)), NaCl (200 mM), glycerol (20%), trehalose (250 mM)). The eluted fractions containing LmbB2 were analyzed by SDS PAGE. LmbB2 was then transferred to buffer D (glycine buffer (80 mM, pH 9.0)), NaCl (200 mM), glycerol (20%), trehalose (250 mM)) with the aid of a 5-mL HiTrap Desalting column (Amersham Pharmacia Biotech, Uppsala, Sweden), concentrated using Centricon YM-10 (Millipore, USA)and immediately used in experiments.

### Assay of tyrosine hydroxylating activity

The amount of DOPA produced was determined as described previously [46]. The assay was performed in triplicates with a control sample containing the respective buffer instead of the enzyme.

### Enzymatic synthesis of DOPA

The LmbB2 reaction product with the retention time corresponding to the DOPA standard was purified by RP-HPLC as described previously [46] and fractions containing the LmbB2 reaction product were pooled. Mass spectrometry was performed on a commercial APEX-Qe FTMS instrument equipped with a 9.4 T superconducting magnet (Bruker Daltonics, Billerica, MA). The cell was opened for 1.3 msec, accumulation time was set at 0.2 s, and one experiment was performed with each sample. One experiment consisted of the average of four spectra. The acquisition data set size was set to 1M points with the mass range starting at *m/z* 150 a.m.u., resulting in a resolution of 100000 at *m/z* 400. The instrument was externally calibrated using clusters of arginine resulting in mass accuracy below 1 ppm. The acquired spectra were apodized with a square sine bell function and Fourier transformed with one zero-fill. The results of mass spectra were interpreted using DataAnalysis version 4.0 software package (Bruker Daltonics, Billerica MA).

### Ferrous thiocyanate method for determination of H_2_O_2_

The formation of H_2_O_2_ by LmbB2 was determined by the ferrous thiocyanate assay [47] modified for use in clear flat bottom 96-well plates [48]. Authentic H_2_O_2_ diluted in the respective buffer and carried through the assay procedures was used to record calibration curves which were linear from 1 to 100 µM H_2_O_2_. Blank values were determined in the absence of the enzyme but with all other buffer components present.

The freshly purified LmbB2 (10–1000 µg; approx. 10% heme occupation) in buffer D was used in the assay. Measurements were performed in duplicates.

### Spectral studies

Light-absorption spectra of purified MBP2*-LmbB2 were recorded with a HP8453E Spectroscopy System between 300–700 nm. The spectra were obtained at room temperature using a 1-cm quartz cuvette containing either native or with a few grains of sodium dithionite reduced enzyme dissolved in buffer A containing maltose (10 mM). Baselines were routinely subtracted from spectra.

*Sodium dithionite-reduced carbon monooxide (CO) difference spectra -* The spectra were obtained on an HP8453E Spectroscopy System. The reduced CO spectrum of purified MBP2*-LmbB2 was obtained after bubbling CO through a solution of MBP2*-LmbB2 protein reduced with a few grains of sodium dithionite dissolved in buffer A containing maltose (10 mM) for several minutes.

RRS spectra were obtained using a multichannel Raman spectrograph (Jobin Yvon–Spex 270 M) equipped with a holographic notch-plus filter (Kaiser) and a liquid nitrogen-cooled CCD detector (Princeton Instruments). Soret band excitation at 441.6 nm provided by a He-Cd laser (Liconix) with the power at the sample of ∼5 mW was used at 90° scattering geometry. Measurements were carried out at 20°C using a stationary temperature-stabilized microcell (10 µL). Wavenumber scales of RRS spectra were precisely calibrated using spectra of a neon glow lamp taken before and after each Raman measurement. RRS spectra were recorded as series of 10–30 separate frames (30 s accumulation per frame) to disclose possible spectral changes during acquisition. Strong fluorescence background superimposing RRS spectra were subtracted using a polynomial approximation.

### Cofactor preparation and MS analysis

#### Analytical mode (UPLC)

An Acquity UPLC system (Waters, Milford, Massachusetts), equipped with 2996 PDA detector operating at 400 nm was used for analysis. Data were processed with Empower 2 software (Waters). Samples were analyzed on a Waters BEH C18 column (50 mm×2.1 mm I.D, particle size 1.7 µm), column temperature 20°C; data sample rate 20 pts/s; filter constant 0.5; injection volume 5 µL; analysis time 10 min; flow rate 0.4 mL min^−1^. Mobile phases consisted of water (A) and acetonitrile (B), both containing trifluoroacetic acid (0.1%). Gradient elution started at 5% B, increasing linearly to 95% B within 10 min. The sample was mixed with acetonitrile and trifluoroacetic acid to a final concentration of 5% and 0.1%, respectively, before loading. Each analysis was followed by an equilibration step (1 min).

#### Semi-preparative mode (HPLC)

HPLC analyses were performed on a Waters system (Waters) equipped with flow controller 600, autosampler 717, and UV detector 486 set at 400 nm. Data were processed with Millennium 32 software (Waters). Analytes were prepared on Waters semi-preparative column XTerra RP18 (150×7.8 mm I.D., particle size 5 µm), protected with Waters XTerra RP18 Guard column (20×3.9 mm I.D., particle size 5 µm). Mobile phases consisted of water (A) and acetonitrile (B), both containing trifluoroacetic acid (0.1%). Gradient elution started at 10% B, increasing linearly to 90% B within 20 min. Each analysis was followed by an equilibration step (15 min).

The fractions exhibiting UV absorption at 398 and 405 nm were collected for MS characterization. Fractions were evaporated to dryness under reduced pressure and subjected to HR-MS.

### DOPA analysis (UPLC)

UPLC analyses were performed in an Acquity UPLC system (Waters, USA), equipped with a 2996 photodiode array detection (DAD) system with detector operating from 194 to 350 nm. Data were processed using the Empower 2 software (Waters). The following chromatographic conditions were applied: BEH C18 column; flow rate, 0.4 ml min^−1^, data sample rate, 20 pts s^−1^; filter constant, 0.5; injection volume, 5 µL. Samples were analyzed by isocratic elution in formic acid (0.08%).

### Circular dichroism (CD) spectroscopy

CD spectra were recorded at 20°C using a Jasco J-810 spectropolarimeter equipped with Peltier temperature controller (Jasco, Tokyo, Japan). Data were collected from 195 to 260 nm, at 100 nm min^−1^, 1 s response time and 2 nm bandwidth using a 0.1-cm quartz cuvette containing studied protein in Britton-Robinson buffer (pH 8.95). Each spectrum shown is the average of ten individual scans and was corrected for absorbance caused by the buffer. CD data were expressed in terms of the mean residue ellipticity (*Θ*_MRE_) using the equation:
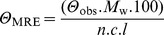
where *Θ*_obs_ is the observed ellipticity in degrees, *M*_w_ is the protein molecular weight (36 477 g mol^−1^), *n* is number of residues (317 aa+his tag of pET42 = 327 aa), *l* is the cell path length (0.1 cm), *c* is the protein concentration (0.15 mg mL^−1^) and the factor 100 originates from the conversion of the molecular weight to mg dmol^−1^.

Thermal unfolding of LmbB2 protein in the presence of different concentrations of BH_4_ was followed by monitoring the ellipticity at 220 nm over the temperature range of 20 to 70°C, with a resolution 0.1°C, at a heating rate 1°C min^−1^. Recorded thermal denaturation curves were fitted to sigmoidal curves using software Origin 6.1 (OriginLab, Massachusetts, USA). The melting temperatures (*T*_m_) were evaluated as a midpoint of the thermal transition.

### Biochemical characteristics of LmbB2

The thermostability of LmbB2 was determined by incubating the enzyme (7 mg mL^−1^) in buffer D for 30 min at various temperatures. The incubated enzyme was allowed to cool to 4°C in 5 min, centrifuged and the tyrosine hydroxylating activity was assayed.

The effect of inhibitors on LmbB2 was determined by assaying the tyrosine hydroxylating activity as described above. The inhibitors SDS (0.01%; 0.02%) and EDTA (0.1 mM; 1 mM) were added from 100× stock solutions.

The effect of reduced cofactors was determined by assaying the tyrosine hydroxylating activity in the presence of the reduced cofactors (NADH, NADPH, BH_4_, tetrahydrofolic acid), added from 100× stock solutions, each at a final concentration of 2 mM.

### Velocity dependence on concentration of tyrosine and BH_4_

The reactions were performed as described in “Assay of tyrosine hydroxylating activity”. Velocity dependence on the concentration of tyrosine and BH_4_ was monitored by varying concentration of one substrate while leaving the concentration of the other substrates fixed. The tyrosine and BH_4_ concentration varied from 1.2 to 1200 µM and 7.8 to 3000 µM, respectively. The concentration of oxygen was kept at a concentration not limiting the LmbB2 reaction.

### Miscellaneous methods

Protein concentration was determined by means of the BCA Protein Assay Reagent kit (Pierce, Rockford, USA) using BSA (Sigma) as standard. SDS/PAGE [49] was performed under reducing conditions in an LKB II - electrophoresis unit (LKB Amersham Pharmacia Biotech, Uppsala, Sweden) and visualized with Coomassie Brilliant Blue R250. Protein sequences were aligned using BLAST and PSI-BLAST [50], [44]. All DNA manipulations were performed as described in Ausubel et al. [51].

## Supporting Information

Figure S1 **Photoreduction of the LmbB2 upon laser irradiation.** (A) Absorption spectra of native oxidized MBP2*-LmbB2 (Ox) before irradiation and after 30 min laser irradiation at 441.6 nm (Ph). Spectral changes characteristic for photoreduction are highlighted in difference absorption spectrum (Ph-Ox). (B) Absorption spectra of oxidized MBP2*-LmbB2 (Ox) before irradiation and after 30 min laser irradiation (Ph). Third spectrum (Reox) was taken from the irradiated sample (Ph) after 30 min relaxation in dark. Changes in the Soret band the Reox spectrum seem to demonstrate reoxidation of MBP2*-LmbB2, nevertheless, some changes inthe Q-band region (e.g. 660 nm) remain.
(TIF)

Figure S2 **(A) UPLC analysis of the LmbB2 chromophore content(s).** The LmbB2 protein after adding acetonitrile and TFA (trifluoroacetic acid) was applied directly on the chromatographic column. The resulting chromatogram was extracted at 400 nm, UV spectra of target peaks I and II are attached. Both the retention time and UV/Vis maxima (peak I −5.2 min, 398.3 nm and peak II −7.0 min, 404.3 nm) were in agreement with those of the respective standards (heme b, protoporphyrin IX). *Chromatographic conditions*: Acquity UPLC BEH C18 column (50×2.1 mm I.D., 1.7 µm), column temperature, 22°C; injection volume, 5 µL; flow rate, 0.4 mL min−1; mobile phase consisted of water (A) and acetonitrile (B), both containing TFA (0.1%); gradient elution started at 5% B, increasing linearly to 95% B within 10 min. Each analysis was followed by equilibration step (1 min). **(B) HR-MS analysis of the HPLC purified LmbB2 chromophores.** Analysis of the fractions I and II corresponding to the peaks I and II from Supporting information 2A bears witness of their heme b (*m/z* = 616.17687) and protoporphyrin IX (*m/z* = 563.26489) identity, respectively.
(TIF)

Figure S3 **(A) UPLC analysis of 3,4-DOPA standard (I) and the LmbB2 reaction product (II).** The LmbB2 reaction product was pre-concentrated under HPLC conditions. About 20 fractions were collected and combined, dried under vacuum, reconstituted in formic acid (0.08%), analyzed under UPLC conditions and compared with the 3,4-DOPA standard. Retention time and extracted UV spectrum of this compound (A II) were compared with those of the 3,4-DOPA standard (A I). The identity of 3,4-DOPA stoichiometric isomer was determined by the absolute UV spectra conformity of 3,4-DOPA standard (A I) and the LmbB2 reaction product (A II). **(B) HR-MS analysis of the HPLC purified LmbB2 reaction product.** HR-MS spectrum of the DOPA standard (I) showed identical peak at *m/z* of 198.07615 as the HR-MS spectrum of purified LmbB2 reaction product (II).
(TIF)

Figure S4 **Temperature stability of LmbB2.** Temperature stability of LmbB2 was determined by incubating the enzyme (7 mg mL^−1^) in buffer D (glycine buffer (80 mM, pH 9.0)), NaCl (200 mM), glycerol (20%), trehalose (250 mM)) for 30 min at variol temperatures. The incubated enzyme was allowed to cool to 4°C in 5 min, centrifuged and subjected to the tyrosine hydroxylating activity assay at 25°C.
(TIF)

Table S1 **Sequences producing significant alignments with LmbB2.**
(TIF)
