# Supplementary figures and images for: Lincomycin Biosynthesis Involves a Tyrosine Hydroxylating Heme Protein of an Unusual Enzyme Family
Source: PLoS One. 2013 Dec 4;8(12):e79974. doi: 10.1371/journal.pone.0079974 (PMC3851162; doi:10.1371/journal.pone.0079974)

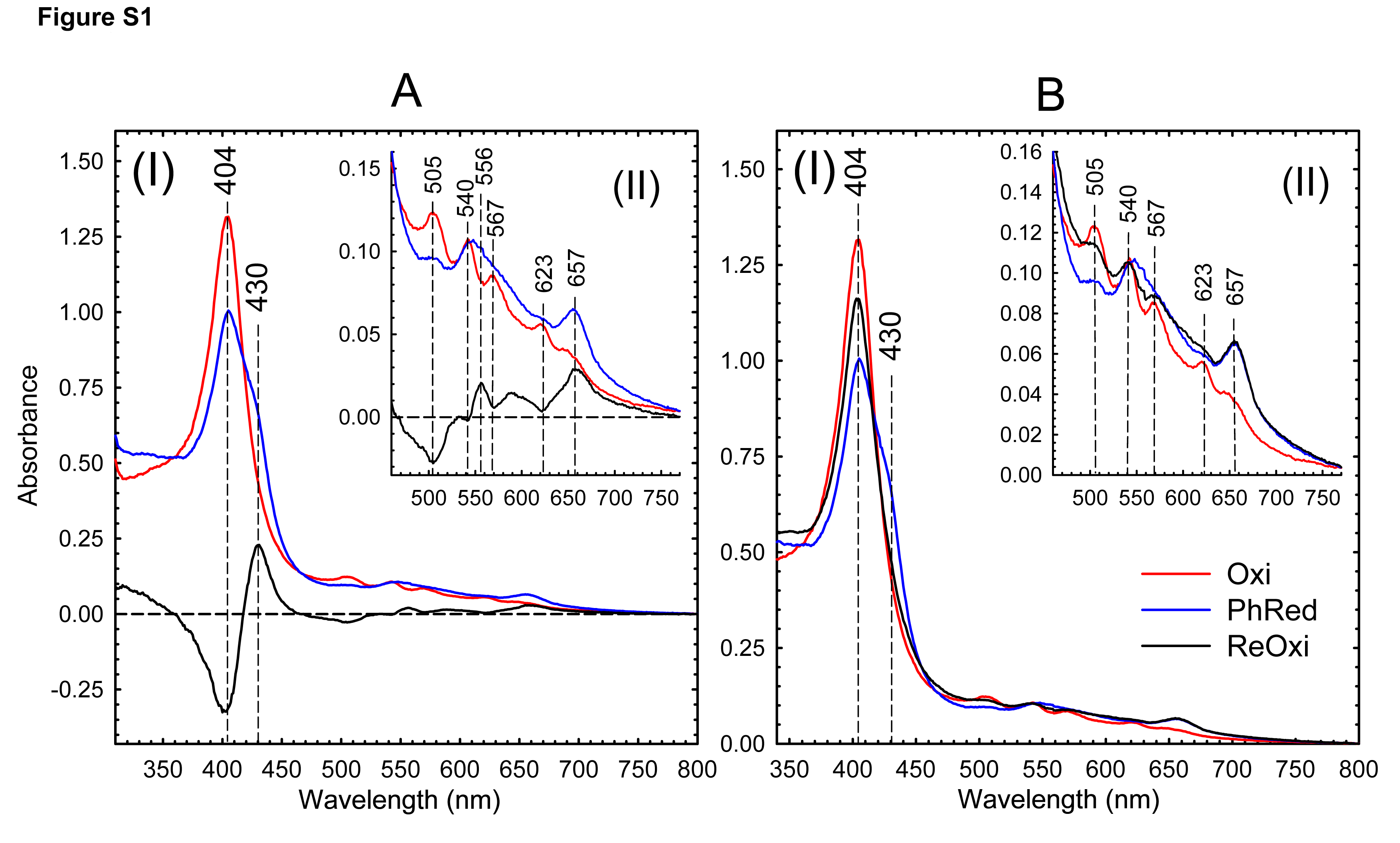

Supplement: Figure S1 — Photoreduction of the LmbB2 upon laser irradiation. (A) Absorption spectra of native oxidized MBP2*-LmbB2 (Ox) before irradiation and after 30 min laser irradiation at 441.6 nm (Ph). Spectral changes characteristic for photoreduction are highlighted in difference absorption spectrum (Ph-Ox). (B) Absorption spectra of oxidized MBP2*-LmbB2 (Ox) before irradiation and after 30 min laser irradiation (Ph). Third spectrum (Reox) was taken from the irradiated sample (Ph) after 30 min relaxation in dark. Changes in the Soret band the Reox spectrum seem to demonstrate reoxidation of MBP2*-LmbB2, nevertheless, some changes inthe Q-band region (e.g. 660 nm) remain. (TIF) [file pone.0079974.s001.tif]

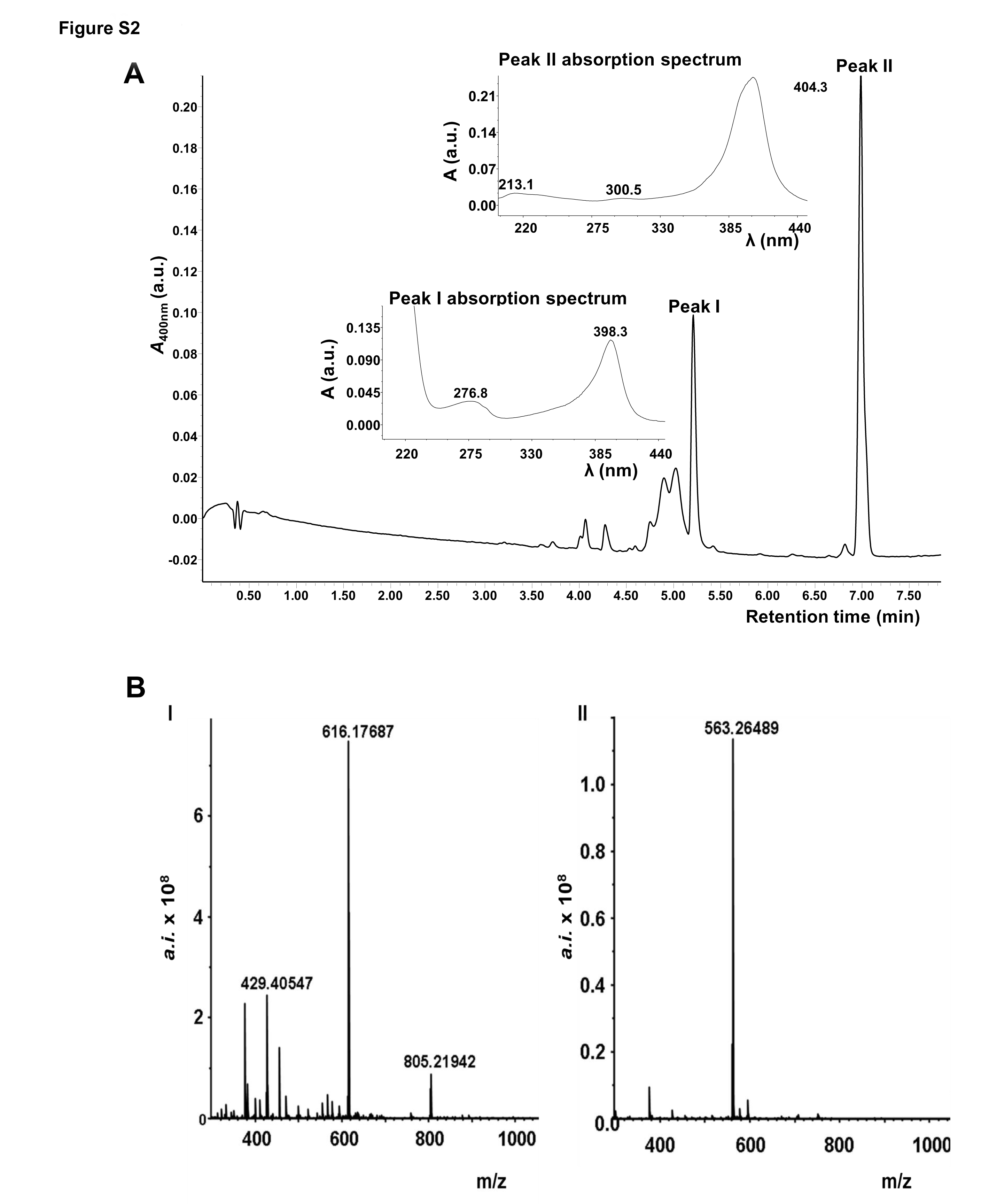

Supplement: Figure S2 — (A) UPLC analysis of the LmbB2 chromophore content(s). The LmbB2 protein after adding acetonitrile and TFA (trifluoroacetic acid) was applied directly on the chromatographic column. The resulting chromatogram was extracted at 400 nm, UV spectra of target peaks I and II are attached. Both the retention time and UV/Vis maxima (peak I −5.2 min, 398.3 nm and peak II −7.0 min, 404.3 nm) were in agreement with those of the respective standards (heme b, protoporphyrin IX). Chromatographic conditions: Acquity UPLC BEH C18 column (50×2.1 mm I.D., 1.7 µm), column temperature, 22°C; injection volume, 5 µL; flow rate, 0.4 mL min−1; mobile phase consisted of water (A) and acetonitrile (B), both containing TFA (0.1%); gradient elution started at 5% B, increasing linearly to 95% B within 10 min. Each analysis was followed by equilibration step (1 min). (B) HR-MS analysis of the HPLC purified LmbB2 chromophores. Analysis of the fractions I and II corresponding to the peaks I and II from Supporting information 2A bears witness of their heme b (m/z = 616.17687) and protoporphyrin IX (m/z = 563.26489) identity, respectively. (TIF) [file pone.0079974.s002.tif]

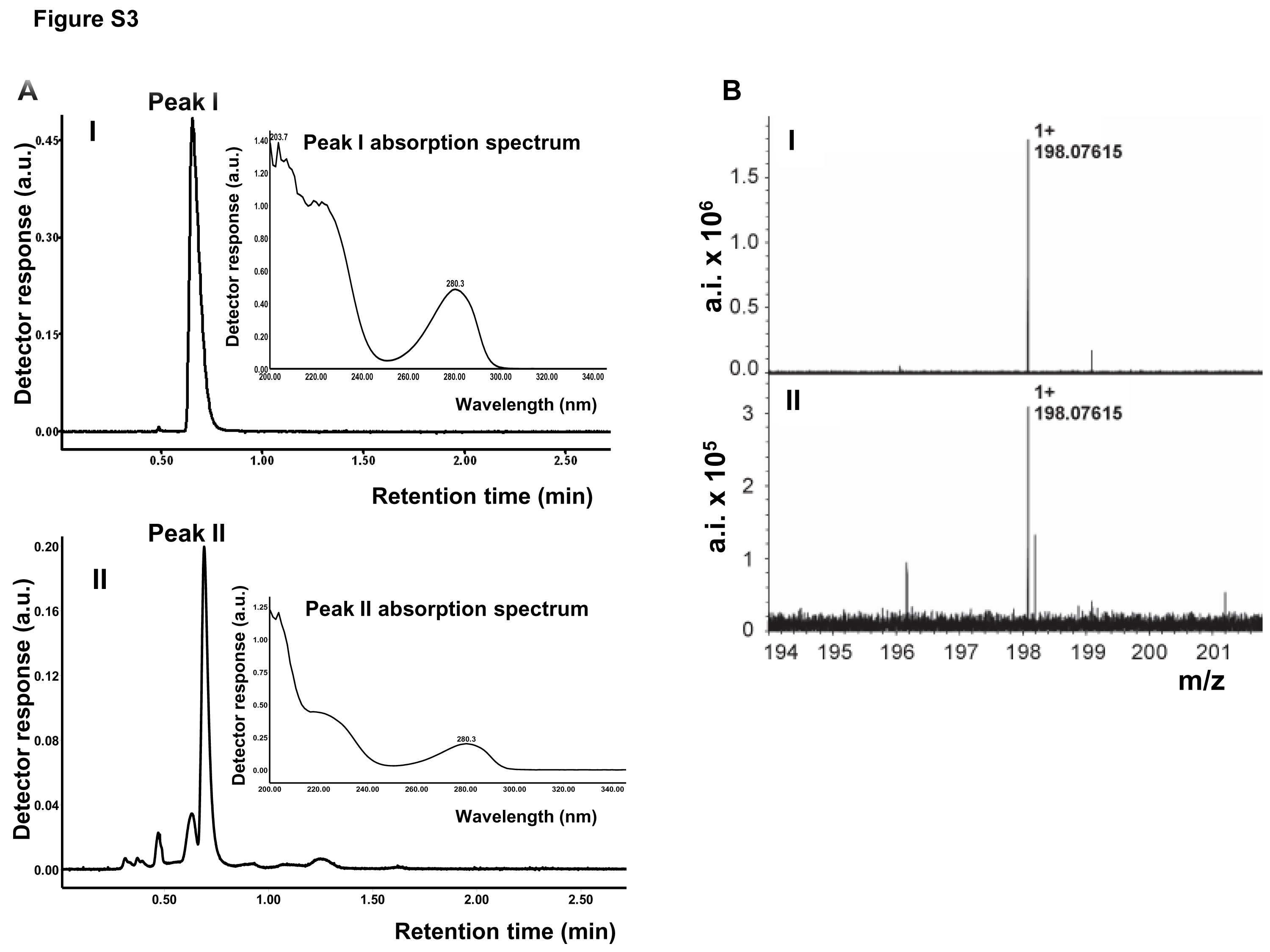

Supplement: Figure S3 — (A) UPLC analysis of 3,4-DOPA standard (I) and the LmbB2 reaction product (II). The LmbB2 reaction product was pre-concentrated under HPLC conditions. About 20 fractions were collected and combined, dried under vacuum, reconstituted in formic acid (0.08%), analyzed under UPLC conditions and compared with the 3,4-DOPA standard. Retention time and extracted UV spectrum of this compound (A II) were compared with those of the 3,4-DOPA standard (A I). The identity of 3,4-DOPA stoichiometric isomer was determined by the absolute UV spectra conformity of 3,4-DOPA standard (A I) and the LmbB2 reaction product (A II). (B) HR-MS analysis of the HPLC purified LmbB2 reaction product. HR-MS spectrum of the DOPA standard (I) showed identical peak at m/z of 198.07615 as the HR-MS spectrum of purified LmbB2 reaction product (II). (TIF) [file pone.0079974.s003.tif]

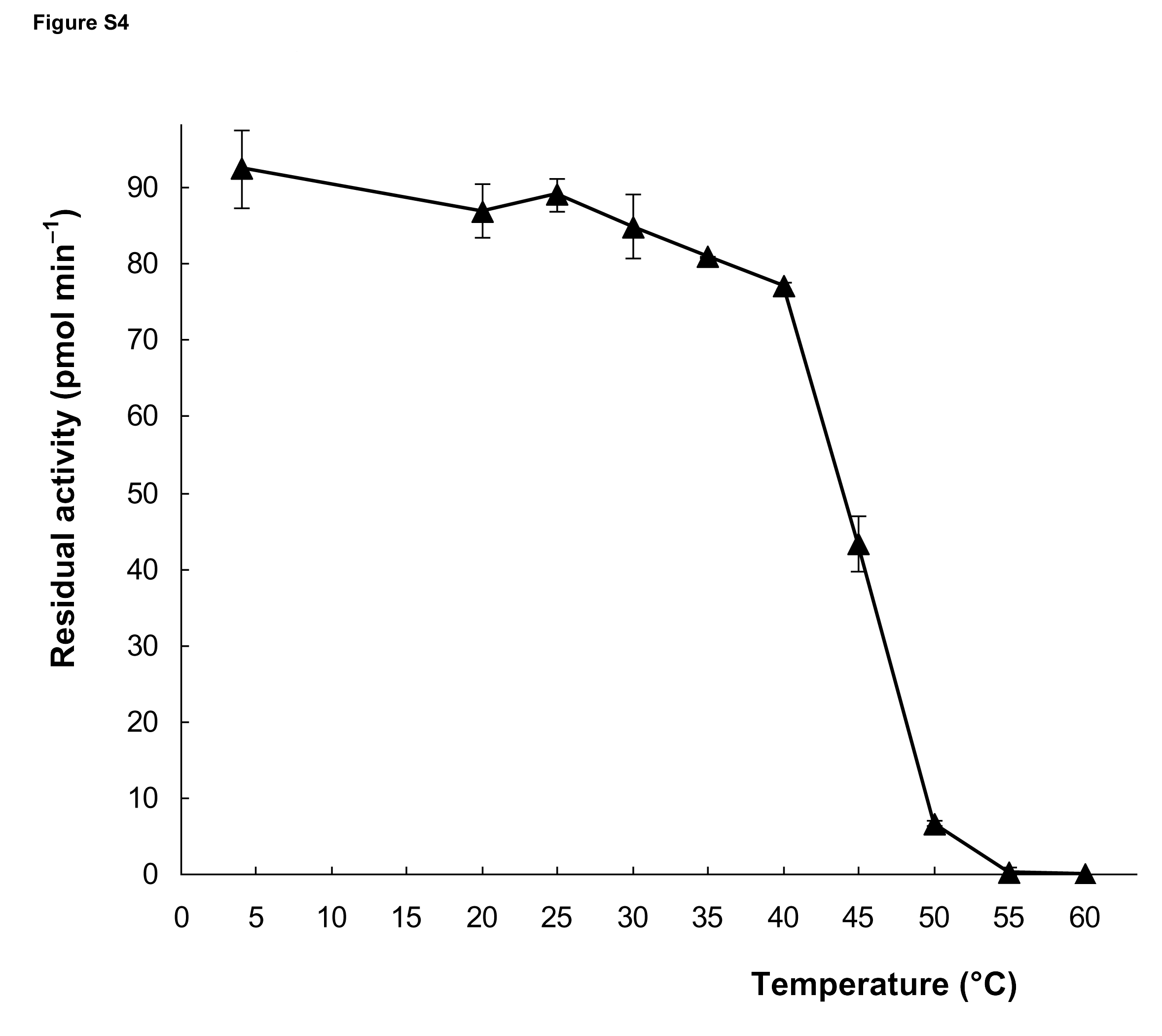

Supplement: Figure S4 — Temperature stability of LmbB2. Temperature stability of LmbB2 was determined by incubating the enzyme (7 mg mL−1) in buffer D (glycine buffer (80 mM, pH 9.0)), NaCl (200 mM), glycerol (20%), trehalose (250 mM)) for 30 min at variol temperatures. The incubated enzyme was allowed to cool to 4°C in 5 min, centrifuged and subjected to the tyrosine hydroxylating activity assay at 25°C. (TIF) [file pone.0079974.s004.tif]

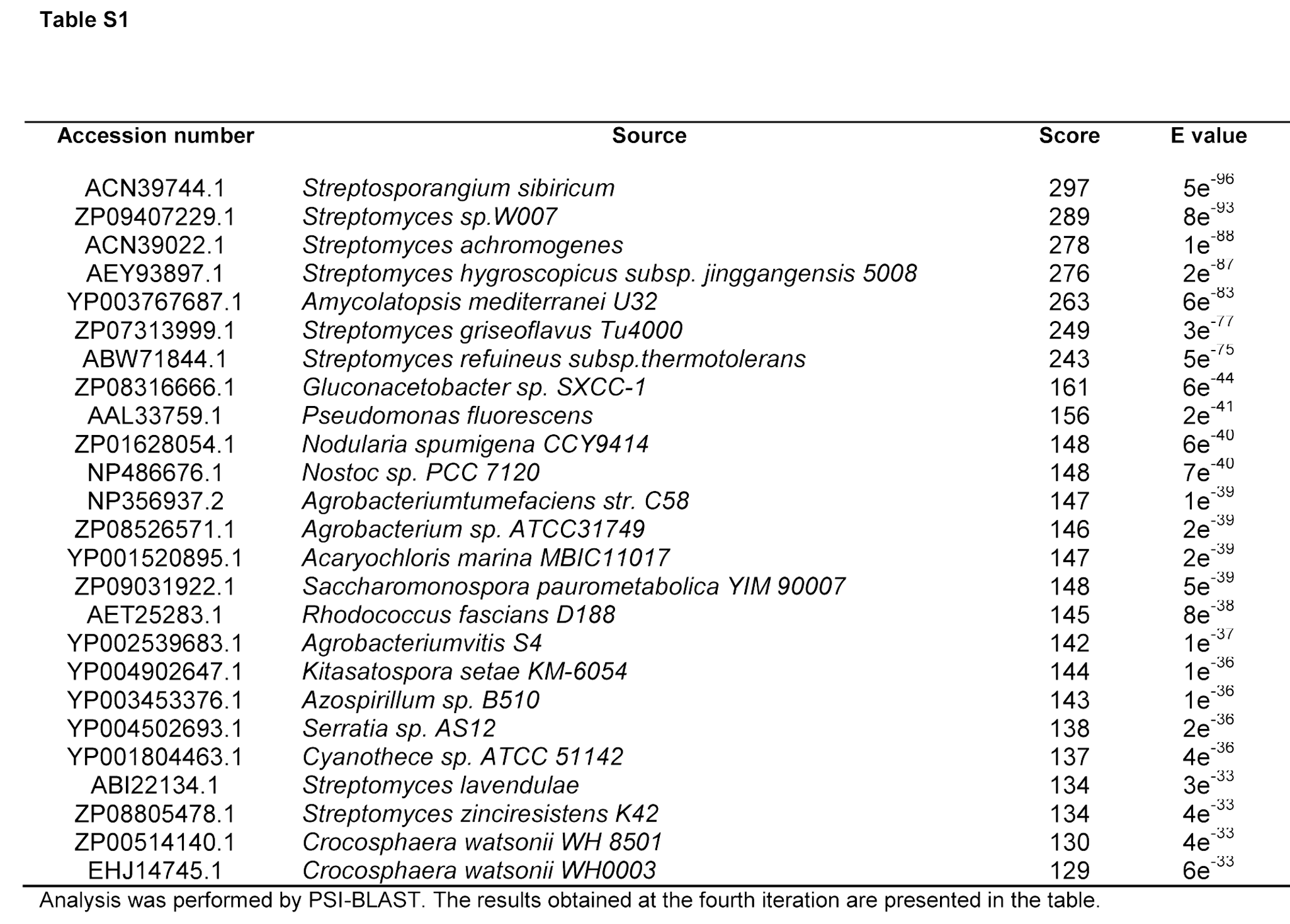

Supplement: Table S1 — Sequences producing significant alignments with LmbB2. (TIF) [file pone.0079974.s005.tif]
